# Plant traits, productivity, biomass and soil properties from forest sites in the Pacific Northwest, 1999–2014

**DOI:** 10.1038/sdata.2016.2

**Published:** 2016-01-19

**Authors:** Logan T. Berner, Beverly E. Law

**Affiliations:** 1 Department of Forest Ecosystems and Society, Oregon State University, 321 Richardson Hall, Corvallis, Oregon 97331, USA.

**Keywords:** Ecological modelling, Environmental chemistry, Forestry, Plant physiology, Biogeochemistry

## Abstract

Plant trait measurements are needed for evaluating ecological responses to environmental conditions and for ecosystem process model development, parameterization, and testing. We present a standardized dataset integrating measurements from projects conducted by the Terrestrial Ecosystem Research and Regional Analysis- Pacific Northwest (TERRA-PNW) research group between 1999 and 2014 across Oregon and Northern California, where measurements were collected for scaling and modeling regional terrestrial carbon processes with models such as Biome-BGC and the Community Land Model. The dataset contains measurements of specific leaf area, leaf longevity, leaf carbon and nitrogen for 35 tree and shrub species derived from more than 1,200 branch samples collected from over 200 forest plots, including several AmeriFlux sites. The dataset also contains plot-level measurements of forest composition, structure (e.g., tree biomass), and productivity, as well as measurements of soil structure (e.g., bulk density) and chemistry (e.g., carbon). Publically-archiving regional datasets of standardized, co-located, and geo-referenced plant trait measurements will advance the ability of earth system models to capture species-level climate sensitivity at regional to global scales.

## Background & Summary

Earth system models (ESMs) play an important role in climate change mitigation and adaptation efforts, enabling evaluation of potential future climate impacts and management decisions on ecological systems; however, these models have typically been parameterized with generalized Plant Functional Types (PFTs), which makes it difficult to predict species impacts and shifts within regions. Parameterizing ESMs with species or genus-level traits is more desirable, yet model development, testing and applications have been limited by the availability of sufficient field measurements for regional to global modeling^1^. Thus, there is a pressing need for standardized, spatially-extensive measurement of select plant traits and associated plot-level characteristics.

**Figure 1:**
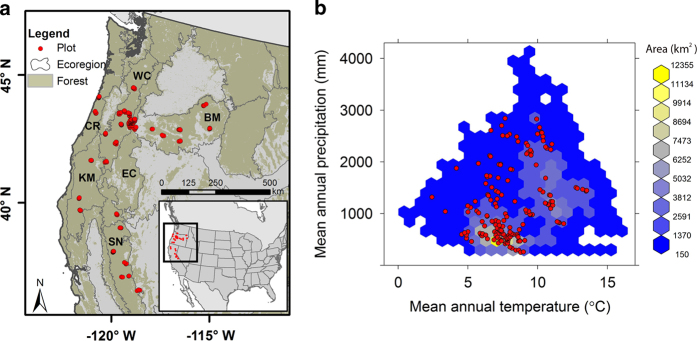
Location of sampling sites in (**a**) geographic space and (**b**) climate space. Sampling sites (*n*=239) were spread among six forested ecoregions located in Oregon and northern California. The sampling sites covered much of the climate space encounter across the forested parts of the ecoregions. Only climate spaces occupying at least 150 km^2^ are shown in the figure. Climate data were from PRISM and were averaged from 1984 to 2013.

We have developed a dataset that includes ESM-relevant leaf trait measurements (specific leaf area, nitrogen, carbon, lifespan) for 35 tree and shrub species from 239 sites in Oregon and northern California (Fig. 1a,b). The dataset also includes plot-level characteristics (e.g., biomass, productivity, soil depth) for the sites. The dataset incorporates measurements from projects in the Pacific Northwest from 1999–2014 that focused on evaluation of ecosystem processes and model development and testing (Table 1). Protocols for field sampling, laboratory analysis, computations, and data submission were developed and implemented in 1999-2000 (ref. 2). Below we provide a brief description of each research project.

The RADAR project (1999) focused on the East Cascades and sought to characterize forest canopy structure and biomass, as well as develop and test methods for estimating biomass from fusion of radar and AVIRIS hyperspectral imagery^3,4^. Measurements were made on 20 plots, largely dominated by ponderosa pine (*Pinus ponderosa*), but several or grand fir (*Abies grandis*). The leaf area index (LAI) sampling was more intensive than the standard protocol^2^ given the focus on 3-dimensional canopy modeling^5,6^.

The EPA project (2001) was a regional study over Oregon and northern California that aimed to quantify current biomass and net ecosystem production (NEP) by integrating remote sensing, intensive plots, extensive plots, inventories and modeling using a spatially nested hierarchical design^2,7^. Measurements were made on 96 plots, with 36 plots spread among three intensive clusters (Coast Range, West Cascades, East Cascades), each of which included 4 age classes x 3 replications. The remaining 60 plots were distributed regionally to capture Landsat spectral variability.

The COHO project (2002) focused on measuring and modeling carbon stocks and fluxes at five sites it the East Cascades using biometric and eddy covariance measurements in conjunction with the Soil-Plant-Atmosphere (SPA) model^8^. Measurements were made at young, mature and old ponderosa pine sites, as well as at mature grand fir and western juniper (*Juniperus occidentalis*) sites.

The Oregon and California (ORCA) project (2004–2005) sought to broaden the regional sampling network and tree species representation though extensive sampling in the East Cascades, Blue Mountains, Klamath Mountains, northern Great Basin, and Sierra Nevada, with measurements made on 80 plots^9,10^. In 2005, measurements were made on 14 of these plots in the Sierra Nevada as part of the Forest Hill thinning study, where the impacts of thinning on productivity and carbon allocation by trees and shrubs were assessed both 3- and 16-years after thinning in relation to unthinned plots.

The Metolius Fire (METOFIRE) project (2007–2008) focused on quantifying pre- and post-fire carbon pools and productivity on four mixed-severity wildfires (2002–2003) in mixed fir and ponderosa pine forests in East Cascades^11^. Measurements were made on 64 plots, with burned and unburned stands measured in 2007 and 2008, respectively.

The Cascade Drought Impact Study (CADIS; 2014) evaluated the role of water availability in shaping tree morphological traits and forest carbon cycling along a steep climatic gradient in the East Cascades^12^. Measurements were made on 15 sites spread evenly among western juniper, ponderosa pine, and grand fir.

Portions of this dataset have furthermore been used to (1) parameterize and test ecological models (e.g., Biome-BGC^13^, CLM^14^); (2) evaluate satellite algorithms^15^ and eddy covariance measurements^16^; (3) assess regional carbon budgets^10^ and consequences of forest management (e.g., harvest regimes^13,17^, bioenergy production^18,19^); and (4) explore relationships between soil carbon and detritus^20^. We are confident that the research community will find additional uses for this dataset. This article introduces the dataset and associated methods, describes each variable, and provides statistical summaries of the leaf traits by species, and summaries of the remaining variables (e.g., biomass, productivity, soil characteristics) at the plot level.

## Methods

This section provides brief descriptions of the field and laboratory measurement protocols, as well as of the leaf, stand, and soil variables included in the dataset. The field, lab and computational methods are described in detail in an FAO protocol document that was subsequently developed for the AmeriFlux network and FLUXNET^2^.

### Field sampling

The plot design consisted of a 1 ha plot containing four subplots (center, north, southwest, southeast) that were spaced at 35 m between subplot centers, with subplot diameter ranging from 10 to 17 m, depending on tree density. Tree height and diameter at breast height (DBH) were measured on each subplot for all stems that were 10–80 cm DBH. All large trees >80 cm DBH were measured on the entire 1 ha plot, while sapling surveys (stems 1–10 cm DBH) were conducted inside the subplots with radii of 2–5 m depending on tree density. Around twenty tree cores were collected per plot, with half used to estimate annual growth and age, and the remaining used to determine wood density. Furthermore, overstory leaf area index (LAI, one-sided) was measured at 35–45 locations on each subplot using an LAI-2000 or LAI-2200 (LiCor, Lincoln, NE).

Mid-canopy, south-facing branches with high, but vertically-variable light exposure were harvested for each species present on a plot, with sample size per species (generally 5 to 10) determined by the species prevalence within a plot. Leaf samples from this canopy position tend to be a reasonable approximation for canopy-average trait characteristics^21^, which are necessary in most ecosystem models. Additionally, soil cores were collected from multiple layers (e.g., 0–20, 20–50, 50–100 cm).

### Foliage analysis

#### Leaf carbon and nitrogen content

**Figure 2:**
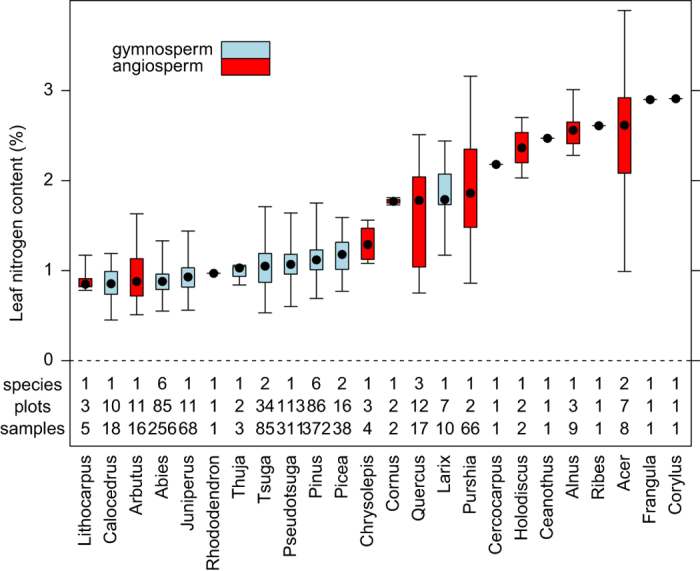
Box and whisker plots summarizing leaf nitrogen measurements for each plant genera represented in the dataset. Intrabox dots denote medians, box edges denote 25th and 75th percentiles, and whiskers denote minimum and maximum values. The number of species, sites, and samples representing each genera is also provided. Species-level and genus-level statistical summaries are given in Tables 2 and 3.

Leaf carbon or nitrogen content per unit of dry mass, expressed as a percentage of leaf dry mass. Leaf carbon and nitrogen content were measured on one-year old foliage, except in the case of deciduous species, where measurements were based on current-year, fully-expanded and hardened foliage. Leaf samples were oven-dried at 70 °C for at least 48 h and then finely ground using a coffee-grinder and/or mortar and pestle. Carbon and nitrogen concentrations were then measured with a LECO CNS2000 analyzer by Central Analytical Laboratory at Oregon State University. Genus-level graphical summaries of leaf nitrogen are presented in Fig. 2, while species-level and genus-level statistical summaries of both leaf carbon and nitrogen are given in Tables 2 and 3.

#### Specific leaf area

**Figure 3:**
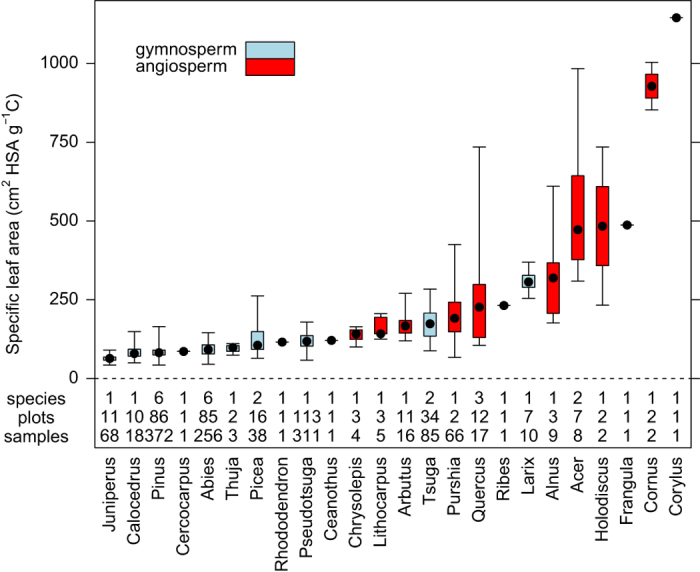
Box and whisker plots summarizing specific leaf area measurements for each plant genera represented in the dataset. Intrabox dots denote medians, box edges denote 25th and 75th percentiles, and whiskers denote minimum and maximum values. The number of species, sites, and samples representing each genera is also provided. Species-level and genus-level statistical summaries are given in Tables 2 and 3.

Specific leaf area is the ratio of leaf surface area to carbon mass. We provide SLA estimates calculated using both leaf projected surface area (PSA; cm^2^ PSA g^−1^ C) and leaf hemi-surface area (i.e., one-half total leaf area; HSA; cm^2^ HSA g^−1^ C). As with leaf chemistry, specific leaf area was measured on one-year old foliage, except in the case of deciduous species, where measurements were based on current-year, fully-expanded and hardened foliage. Fresh leaf PSA was measured using a LI-3100C Area Meter (LiCor, Lincoln, NE). For broad-leaf angiosperms, leaf PSA was assumed to be equivalent to HSA; while for conifers, leaf PSA was converted to HSA using published conversion coefficients^22–26^ (Table 4). Species with unknown conversion coefficients were assigned values from similar species. For *Pinus* species, leaf HSA was estimated from measurements of needle length and maximum fascicle diameter, except for samples collected as part of the CADIS project, which were scanned using the LI-3100C. After measuring leaf surface area, the samples were oven-dried at 70 °C for at least 48 h and then weighed. Leaf dry mass was converted to carbon based on elemental analysis of sample carbon content (described above). The SLA of each sample was then calculated by dividing leaf PSA and/or HSA by carbon mass. Genus-level graphical summaries of SLA are presented in Fig. 3, while species-level and genus-level statistical summaries are given in Tables 2 and 3.

#### Leaf lifespan

Leaf lifespan, also known as leaf longevity or leaf retention time, describes the number of years that a leaf is alive. Leaf lifespan was estimated using the ‘counting cohorts’ method, which involves counting the number of annual leaf cohorts present on an individual branch back to the point where less than 50% of the leaves produced during a given year still remain attached to the branch^27^. Species-level and genus-level statistical summaries of leaf lifespan are given in Tables 2 and 3.

### Stand characterization

#### Geographic location

The latitude, longitude, and elevation are provided for each plot. Geographic coordinates are given in decimal degrees based on the WGS84 geographic datum and elevation is in meters above sea level. Geographic coordinates were determined using a hand-held global positioning system (GPS). Elevations were extracted from the Shuttle Radar Topography Mission version 2 digital elevation model based on the geographic coordinates.

#### Stand age

Stand age is defined in this case as the average age, given in years, of the oldest 10% of trees located in a stand or, if fewer than three trees fell into the oldest 10%, then the average age of all trees that were cored. Stand age was estimated based on tree cores collected from eight to 20 trees on each plot, with sample size dependent on project. Trees were cored to the pith at breast height using an increment borer. The tree cores were mounted on a wooden block and sanded to reveal the annual rings. The age of each tree was then determined by either examining the tree core under a microscope, or by scanning the tree core with a flatbed scanner and then ingesting the image into the WinDENDRO software (Regent Instruments Inc., Québec). For large trees where increment core samples did not reach the pith, the missing rings were determined from total number of rings within 5 cm distance of the inner end of core sample and estimates of the missing length by subtracting increment core length from the radius of the tree at breast height.

#### Species overstory composition

Species overstory composition described the relative dominance of overstory species on a plot and is provided for up to four species. Each species is denoted using a six-letter genus-species abbreviation. Species dominance is described as the percentage of stand basal area represented by the species. Basal area was computed for each tree based on measurement of DBH and then summed within species.

#### Average canopy height

Average canopy height, given in meters, of all trees located on a plot. Tree height was measured using a laser.

#### Leaf area index

Summer maximum leaf area index (LAI; m^2^ leaf m^−2^ ground) of the overstory canopy corrected for canopy and foliage clumping, as well as wood interception^6,28^. Optical measurements of the effective overstory canopy LAI were made at breast height (1.37 m) with a LAI-2000 or LAI-2200 Plant Canopy Analyzer (LI-COR Inc., Lincoln, NE) under diffusive light at 35–45 locations on each sample plot. Measurements were corrected for clumping and wood interception based on the method described in Law *et al.*^26^. The measurements were subject to post-collection processing to remove data points measured below the threshold of irradiance (<0.2 umol m^−2^ s^−1^) of the above canopy sensor. Species-specific values of the needle-to-shoot ratio for foliage clumping within shoot (*gamma*) were from published data of Law *et al.*^26^, Gower *et al.*^29^, and Frazer *et al.*^30^. Species with unknown *gamma* were assigned values from morphologically similar species. In mixed-species stands, the *gamma*-clumping corrections were weighted by the frequency distribution of stem counts of each species, or by the relative basal area (BA; m^2^ wood ha^−1^) of each species, depending on project. Elemental clumping index (*omega*), which quantifies the effect of foliage clumping at scales larger than the shoot, was determined from continuous measurements with a TRAC (3rd Wave Engineering, Ontario, Canada) optical device along three 100-m transects^26^ or by LAI-2200 measurements at each sampling point. Wood interception (*W*), defined here as half-surface area of stem and branches above breast-height (1.37 m), was computed as a function of stand basal area at breast height as

$$W=2.061\times \left(1-e^{\left(-0.006\times BA\right)}\right)$$

based on the strong relationship (r^2^=0.90) observed between *W* and basal area across 96 sites measured as part of the EPA project (Law, unpublished data). Overstory LAI was then computed by subtracting *W* from clumping-corrected LAI.

#### Tree aboveground biomass

Aboveground biomass of live tree wood (i.e., stem, branches, and bark) and tree foliage per square meter of ground (g C m^−2^ ground), as well as total (i.e., wood+foliage) aboveground biomass pool, were estimate for each plot. For each tree on a plot, wood component biomass was estimated based on measurements of tree DBH and height. Stem volume was estimated using species- and ecoregion-specific volume allometric equations; where species- or ecoregion-specific equations were not available, substitutions were made based on growth form and plant type^4,31–33^. Stem volume was then converted to mass based on species-specific wood density, with wood density either measured using tree cores from the plots or taken from regional^34,35^ or national^36^ technical reports prepared by the USDA Forest Service. Branch and bark mass were estimated using species-specific biomass equations, again substituting equations for similar species were necessary. Stem, branch and bark dry mass were assumed to be 51% carbon. Aboveground tree wood biomass was then derived by summing component carbon pools across trees on a subplot, dividing by subplot area, and then averaging across subplots. Tree foliage biomass was estimated for each subplot from overstory LAI and species biomass-weighted average leaf mass per unit area (LMA=1/SLA), converted to carbon mass based on plot-average leaf carbon concentrations, and then averaged across subplots. Total aboveground biomass on each subplot was computed as the sum of wood and foliage biomass.

#### Tree annual aboveground net primary productivity

Annual aboveground net primary productivity of tree wood and tree foliage per square meter of ground per year (g C m^−2^ ground year^−1^), as well as total aboveground productivity, were estimate for each plot. For each tree on a plot wood component net productivity was estimated based on the difference in biomass at two points in time divided by the number of intervening years, generally 5- or 10-years depending on project. Current wood biomass was estimated as described above, while prior wood biomass was estimated by hindcasting tree DBH and height using tree core increment measurements and DBH-height relationships. Wood component productivity was summed across trees on a subplot, divided by subplot area, and then averaged across subplots. Tree foliage productivity was calculated for each subplot by dividing foliage carbon mass, as described above, by the species biomass-weighted average leaf longevity and then averaged across subplots.

### Soil characterization

#### Soil depth

Effective soil depth (cm) measured with a steel tile probe to a maximum depth of 100 cm.

#### Soil profile minimum and maximum depth

Each soil characteristic described below was derived for a given portion of the mineral soil profile, defined by a minimum and maximum depth below the mineral soil surface, given in centimetres. Target depth in each soil profile included 0–20 cm, 20–50 cm, and 50–100 cm; however, the actual profile depths are noted for each layer.

#### Soil bulk density

Soil bulk density for a specified soil profile layer given in kilograms of rock-free soil per square meter of earth (kg m^−2^). Bulk density was determined using material <2 mm diameter based on air-dry weight and soil core volumes that excluded coarser fragments.

#### Soil carbon and nitrogen mass

Carbon or nitrogen mass for a specified mineral soil profile layer, given in grams of C or N per square meter of ground (e.g., g C m^−2^). The soil samples were air-dried using a ventilated drying system and then live vegetation and roots were removed. Samples were pulverized, then carbon and nitrogen content were measured with a LECO CNS2000 analyzer by Central Analytical Laboratory at Oregon State University. Carbon and nitrogen content in each mineral soil profile layer were then computed from elemental concentrations, soil bulk density, and sampling depth. See Sun *et al.*^20^ for more details.

#### Soil pH

Soil pH for a specified profile layer was determined using a ratio of soil mass to water of 1:2. The analysis was performed by Central Analytical Laboratory at Oregon State University.

#### Soil sand, silt, and clay content

Mass fraction of sand (0.05–2 mm diameter), silt (0.002–0.05 mm diameter), and clay (<0.002 mm diameter) for a specified profile layer, with values given as a percentage of total air-dry mass excluding fragments >2 mm diameter. The fraction of each particle size class was determined using air-dried soil samples following the specification hydrometer method described in Gee and Bauder^37^. This analysis was performed by Central Analytical Laboratory at Oregon State University.

## Data Records

The dataset (*NACP TERRA-PNW: Forest Plant Traits, NPP, Biomass, and Soil Properties, 1999–2014*) is hosted with other contributions from the North American Carbon Program (NACP) by the Oak Ridge National Laboratory Distributed Active Archive Center for Biogeochemical Dynamics (Data Citation 1). The dataset includes three files in a comma-separated values format (.csv), where the first row below the column names stores the column units. Missing values in each file are denoted by −9999. The file containing leaf trait measurements (NACP_TERRA_PNW_leaf_trait_dataset.csv) is structured such that each non-header row (*n*=1296) contains all measurements from a single plant (tree or shrub), with columns (*n*=28) describing each plants sampling location, taxonomy, and trait characteristics (Table 5). The file containing measurements of stand composition, biomass, and productivity (NACP_TERRA_PNW_forest_biomass_productivity_dataset.csv) is structured such that each row (*n*=266) describes a sampling site, with columns (*n*=32) describing the sites location, species composition, aboveground biomass, and productivity (Table 6). The file structure largely conforms to the Biological, Ancillary, Disturbance and Metadata (BADM) protocol used by AmeriFlux and Fluxnet^2^. In contrast with the preceding two files, the file containing measurements of soil physical and chemical characteristics (NACP_TERRA_PNW_soil_dataset.csv) is structured in a long-format to accommodate multiple sampling depths at some sites (Table 7). In other words, each row (*n*=467) stores measurements from a site x sampling depth combination, while each column (*n*=25) describes the site location and various soil characteristics. All files include a PLOT_ID column that can be used to link measurements across the datasets. Additionally, some sites were sampled more than once as part of separate projects, in which case each resample is a separate entry. The column PLOT_ID_ALT in combination with latitude and longitude can be used to identify sites that were resampled.

This dataset represents over 15 years of intellectual investment. We request that the dataset is cited if used in a paper and, if incorporated into another dataset, that each data value/row includes a comment noting the dataset citation. Additionally, we would appreciate the opportunity to contribute intellectually and as co-authors to research projects that both incorporate this dataset and view it as a substantial contribution.

## Technical Validation

Multiple steps were taken to ensure the technical quality of the dataset. Most importantly, consistent field and laboratory protocols^2^ were employed among projects. Exceptions did occur, such as *Pinus* leaf area estimated using callipers versus a LI-3100C when deriving SLA. The sampling intensity specified by the protocols was designed to achieve a coefficient of variation <20%. Repeat measurements of leaf area using the LI-3100C tended to vary by less than 3%. Additionally, all elemental analysis of leaf and soil carbon and nitrogen were performed by Central Analytical Laboratory, which incorporated periodic measurements of calibration samples and blanks to ensure accuracy. Furthermore, we took special care to standardize and define the units of SLA, which is very important given that there is no standard definition. Values reported in the literature are often ambiguously defined and can be derived from measurements of projected, one-sided, one-half total, and total surface area divided by either leaf dry mass or leaf carbon mass^29^.

After compiling the dataset, we implemented several quality control measures on the leaf trait (SLA, C, N, and lifespan) measurements. We plotted each variable and combinations of variables to identify and correct errors in data entry, as well as to identify and remove potential erroneous measurements. After correcting obvious data entry errors, we then identified leaf trait measurements that exceeded the species-average by more than three s.d. Making two passes through the dataset, we chose to screening branch samples if any trait measurement exceeded this threshold; a criteria for inclusion that struck a balance between the need to remove erroneous measurements, while also maintain the full range of phenotypic plasticity.

To further evaluate the dataset, we then compared plant functional type-average and species-average leaf trait summaries against published trait estimates^22,38–50^ (Table 8). Our estimates of leaf lifespan, leaf nitrogen, and SLA for evergreen needleleaf trees differed, respectively, by +60%, −15%, and −6% in comparison to global estimates for this PFT from the TRY plant-trait data base^45^. Similarly, our estimates for deciduous broadleaf trees differed from TRY estimates by +95%, 0%, and +12% for the same traits. Some of this variation can be attributed to differences in species mixture between our regional dataset and the globally-oriented TRY dataset.

The species-specific comparisons also yielded a range in agreement. Across six species, our estimates of leaf lifespan differed by 0 to 120% in comparison to literature values, with five out of eight comparisons falling within 25% of each other. The smallest discrepancy was for *Pseudotsuga menziesii* and the largest discrepancy for *Pinus contorta*, which exhibited high geographic variability in leaf lifespan (2–17 years). Our estimates of leaf nitrogen content differed from literature values by 0 to 30% among six species, with five of the eight estimates differing by 10% or less. Lastly, across 13 species our estimates of SLA (HSA) differed by 4 to 60% from published values, with 18 of 27 estimates falling within 25%. Differences in species-specific leaf trait estimates between our study and other studies could be due to (1) trait variation along resource gradients^51,52^; (2) differences in plant exposure to short-term stress (e.g., leaf shedding due to drought-stress); (3) differences in the seasonality of sample collection^49,53^; (4) differences in sampling location within the canopy^53,54^; or (5) differences in sample processing methods (e.g., SLA estimated with or without the petiole)^27^.

## Additional Information

**How to cite this article:** Berner, L. T. & Law, B. E. Plant traits, productivity, biomass and soil properties from forest sites in the Pacific Northwest, 1999–2014. *Sci. Data* 3:160002 doi: 10.1038/sdata.2016.2 (2016).

## Supplementary Material



## Figures and Tables

**Table 1:** Summary of TERRA-PNW projects that have been combined into the dataset.

**Project**	**Year**	**Number of…**	**Key references**			
		**Sites**	**Genera**	**Species**	**Branches**	
RADAR	1999	20	—	—	—	Law *et al.*^6^
EPA	2001	90	19	24	562	Law *et al.*^7^
COHO	2002	3	5	5	86	Schwarz *et al.*^8^
ORCA	2004	73	13	18	397	Law et at.^9^
METOFIRE	2007	64	2	2	88	Meigs *et al.*^11^
CADIS	2014	15	4	6	163	Berner & Law^12^
The primary sampling year and key references are provided for each project, as are number of sites, genera, species, and branch samples.						

**Table 2:** Species-level statistical summaries (average, s.d., sample size) of specific leaf area (SLA; cm^2^ HSA g^−1^ C), leaf carbon (%), leaf nitrogen (%), and leaf lifespan (years) for tree and shrub species sampled in Oregon and Northern California.

**Div.**	**Genus**	**Species**	**SLA**			**Leaf C**			**Leaf N**			**Leaf lifespan**		
			**Avg.**	**s.d.**	***N***	**Avg.**	**s.d.**	***N***	**Avg.**	**s.d.**	***N***	**Avg.**	**s.d.**	***N***
angio.	Acer	circinatum	689	263	4	49.58	1.77	4	2.23	0.94	4	1	0	4
angio.	Acer	macrophyllum	406	121	4	47.45	2.30	4	2.81	0.78	4	1	0	4
angio.	Alnus	rubra	326	136	9	51.03	1.07	9	2.56	0.22	9	1	0	9
angio.	Arbutus	menziesii	168	35	16	47.57	2.10	16	0.93	0.31	16	1	0	0
angio.	Ceanothus	velutinus	121	NA	1	51.20	NA	1	2.47	NA	1	1	NA	1
angio.	Cercocarpus	unknown	86	NA	1	46.56	NA	1	2.18	NA	1	NA	NA	0
angio.	Chrysolepis	chrysophylla	137	28	4	49.77	0.34	4	1.30	0.23	4	1	0	4
angio.	Cornus	unknown	928	107	2	39.98	0.13	2	1.77	0.06	2	NA	NA	2
angio.	Corylus	cornuta	1,146	NA	1	48.50	NA	1	2.91	NA	1	1	NA	1
angio.	Frangula	purshiana	488	NA	1	47.70	NA	1	2.90	NA	1	1	NA	1
angio.	Holodiscus	discolor	484	355	2	49.00	0.57	2	2.37	0.47	2	1	0	2
angio.	Lithocarpus	densiflorus	162	36	5	43.14	4.29	5	0.91	0.16	5	3.4	2.1	5
angio.	Purshia	tridentate	202	86	66	50.09	1.20	66	1.93	0.52	66	NA	NA	66
angio.	Quercus	chrysolepis	130	30	7	43.25	2.82	7	0.96	0.15	7	3.6	1.4	7
angio.	Quercus	garryana	586	205	3	46.51	1.59	3	2.10	0.62	3	1	NA	3
angio.	Quercus	kelloggii	260	59	7	45.54	0.78	7	1.95	0.14	7	1	NA	7
angio.	Rhododendron	macrophyllum	116	NA	1	52.30	NA	1	0.97	NA	1	1	NA	1
angio.	Ribes	divaricatum	232	NA	1	48.40	NA	1	2.61	NA	1	1	NA	1
gymno.	Abies	amabilis	87	18	25	52.13	0.65	25	0.89	0.12	25	5.3	1.6	25
gymno.	Abies	concolor	102	18	89	48.08	1.97	89	0.88	0.13	89	8.7	2.6	89
gymno.	Abies	grandis	91	19	102	48.87	1.71	71	0.88	0.16	71	8.2	2.5	97
gymno.	Abies	lasiocarpa	98	13	7	50.59	2.45	7	0.92	0.08	7	10.1	5.0	7
gymno.	Abies	magnifica	73	11	11	48.89	2.10	11	0.90	0.18	11	19.6	4.7	11
gymno.	Abies	procera	72	20	22	51.72	1.66	22	0.85	0.15	22	3.9	2.1	22
gymno.	Calocedrus	decurrens	82	24	18	48.04	1.62	18	0.84	0.21	18	2.5	2.1	1
gymno.	Juniperus	occidentalis	64	9	68	48.86	1.71	68	0.95	0.20	68	NA	NA	0
gymno.	Larix	occidentalis	309	33	10	46.13	2.31	10	1.87	0.37	10	1	0	10
gymno.	Picea	engelmannii	94	30	11	50.01	2.23	11	0.89	0.12	11	7.2	2.5	11
gymno.	Picea	sitchensis	138	47	27	53.02	0.76	27	1.27	0.16	27	4.4	0.8	27
gymno.	Pinus	contorta	83	10	40	50.06	1.67	40	1.06	0.21	40	8	4.1	40
gymno.	Pinus	flexilis	107	5	3	52.90	0.20	3	1.29	0.12	3	NA	NA	0
gymno.	Pinus	jeffreyi	90	27	45	49.56	0.94	45	1.00	0.16	45	6.6	2.5	45
gymno.	Pinus	lambertiana	103	32	6	47.63	0.93	6	1.05	0.08	6	7	1.7	6
gymno.	Pinus	monticola	103	15	11	50.71	1.03	11	1.40	0.23	11	6.5	1.2	11
gymno.	Pinus	ponderosa	81	11	267	50.77	2.17	267	1.15	0.17	267	4.5	1.2	185
gymno.	Pseudotsuga	menziesii	119	24	310	51.40	1.64	253	1.08	0.19	253	5.5	2.0	306
gymno.	Thuja	plicata	95	18	3	51.03	1.99	3	0.98	0.12	3	5	NA	1
gymno.	Tsuga	heterophylla	175	46	82	52.74	0.98	82	1.05	0.23	82	5.2	1.3	82
gymno.	Tsuga	mertensiana	193	61	3	50.87	0.81	3	0.89	0.21	3	5.3	1.5	3
Divisions (Div.) include angiosperms and gymnosperms.														

**Table 3:** Genus-level statistical summaries (average, s.d., sample size) of specific leaf area (SLA; cm^2^ HSA g^−1^ C), leaf carbon (%), leaf nitrogen (%), and leaf lifespan (years) for tree and shrub species sampled in Oregon and Northern California Divisions.

**Div.**	**Genus**	**SLA**			**Leaf C**			**Leaf N**			**Leaf lifespan**		
		**Avg.**	**s.d.**	***N***	**Avg.**	**s.d.**	***N***	**Avg.**	**s.d.**	***N***	**Avg.**	**s.d.**	***N***
angio.	Acer	548	243	8	48.51	2.22	8	2.52	0.86	8	1	0	8
angio.	Alnus	326	136	9	51.03	1.07	9	2.56	0.22	9	1	0	9
angio.	Arbutus	168	35	16	47.57	2.10	16	0.93	0.31	16	NA	NA	0
angio.	Ceanothus	121	NA	1	51.20	NA	1	2.47	NA	1	1	NA	1
angio.	Cercocarpus	86	NA	1	46.56	NA	1	2.18	NA	1	NA	NA	0
angio.	Chrysolepis	137	28	4	49.77	0.34	4	1.30	0.23	4	1	0	4
angio.	Cornus	928	107	2	39.98	0.13	2	1.77	0.06	2	NA	NA	2
angio.	Corylus	1,146	NA	1	48.50	NA	1	2.91	NA	1	1	NA	1
angio.	Frangula	488	NA	1	47.70	NA	1	2.90	NA	1	1	NA	1
angio.	Holodiscus	484	355	2	49.00	0.57	2	2.37	0.47	2	1	0	2
angio.	Lithocarpus	162	36	5	43.14	4.29	5	0.91	0.16	5	3.40	2.07	5
angio.	Purshia	194	73	66	50.10	1.20	66	1.93	0.52	66	1	0	66
angio.	Quercus	264	185	17	44.77	2.31	17	1.57	0.58	17	2.06	1.56	17
angio.	Rhododendron	116	NA	1	52.30	NA	1	0.97	NA	1	1	NA	1
angio.	Ribes	232	NA	1	48.40	NA	1	2.61	NA	1	1	NA	1
gymno.	Abies	92	20	256	49.26	2.30	225	0.88	0.14	225	8.27	3.92	251
gymno.	Calocedrus	82	24	18	48.04	1.62	18	0.84	0.21	18	4	NA	1
gymno.	Juniperus	64	9	68	48.88	1.71	68	0.94	0.18	68	NA	NA	0
gymno.	Larix	309	33	10	46.13	2.31	10	1.87	0.37	10	1	0	10
gymno.	Picea	125	47	38	52.15	1.92	38	1.16	0.23	38	5.21	1.95	38
gymno.	Pinus	84	16	372	50.51	2.04	372	1.13	0.19	372	5.41	2.47	287
gymno.	Pseudotsuga	120	24	310	51.40	1.64	253	1.08	0.19	253	5.42	1.96	306
gymno.	Thuja	95	18	3	51.03	1.99	3	0.98	0.12	3	5.00	NA	1
gymno.	Tsuga	175	46	85	52.68	1.04	85	1.04	0.23	85	5.20	1.27	85
Divisions (Div.) include angiosperms and gymnosperms.													

**Table 4:** Summary of literature-derived coefficients used to convert projected surface area (PSA) to hemisurface area (HSA) when determining specific leaf area.

**Species**	**Coef**	**Source**	**Note**
Abies amabilis	1.09	Barclay and Goodman^22^	Coef for Abies grandis
Abies concolor	1.09	Barclay and Goodman^22^	Coef for Abies grandis
Abies grandis	1.09	Barclay and Goodman^22^	
Abies lasiocarpa	1.09	Smith *et al.*^23^	Interpreted from Fig. 1 and bias-corrected by 0.16 based on systematic offset in comparison of PINPON, PINCON, and PSEMEN from refs 22,26
Abies magnifica	1.09	Barclay and Goodman^22^	Coef for Abies grandis
Abies procera	1.09	Barclay and Goodman^22^	Coef for Abies grandis
Calocedrus decurrens	1.15	Barclay and Goodman^22^	Coef for Tsuga heterophylla
Juniperus occidentalis	1.57	Hicks and Dugas^24^	
Larix occidentalis	1.29	Gower and Norman^25^	Coef for Lairx decidua
Picea engelmannii	1.19	Smith *et al.*^23^	See note for Abies lasiocarpa
Picea sitchensis	1.16	Barclay and Goodman^22^	
Pinus contorta	1.29	Barclay and Goodman^22^	
Pinus flexilis	1.19	Smith *et al.*^23^	See note for Abies lasiocarpa
Pinus jeffreyi	1.18	Law *et al.*^26^	Coef for Pinus ponderosa
Pinus lambertiana	1.18	Law *et al.*^26^	Coef for Pinus ponderosa
Pinus monticola	1.18	Law *et al.*^26^	Coef for Pinus ponderosa
Pinus ponderosa	1.18	Law *et al.*^26^	Coef for Pinus ponderosa
Pseudotsuga menziesii	1.19	Barclay and Goodman^22^	
Thuja plicata	1.14	Barclay and Goodman^22^	
Tsuga heterophylla	1.15	Barclay and Goodman^22^	
Tsuga mertensiana	1.15	Barclay and Goodman^22^	Coef for Tsuga heterophylla

**Table 5:** Summary of each column included in the leaf trait dataset, including column number, column name, data format, data units, and a short description.

**Col.**	**Col. name**	**Format**	**Units**	**Range**	**Description**
1	PROJECT	categorical	—	—	Project name
2	YEAR	numerical	year	2000–2014	Sampling year
3	MONTH	numerical	month	5–10	Sampling month
4	DAY	numerical	day	1–31	Sampling day
5	ECOREGION	categorical	—	—	EPA Level III Ecoregion
6	CLUSTER	categorical	—	—	General sampling area
7	PLOT_ID	numerical	—	1–1,014	Plot identification number
8	LATITIDE	numerical	decimal degree	37.7844–45.9477	Plot latitude (WGS84)
9	LONGITUDE	numerical	decimal degree	−123.9038–−117.1309	Plot longitude (WGS84)
10	LAI_O	numerical	m^2^ m^−2^	0–14.7	Overstory leaf area index
11	HEIGHTC	numerical	m	1–54	Plot-average canopy height
12	PFT	categorical	—	—	Plant function type
13	DIVISION	categorical	—	—	Angiosperm or gymnosperm
14	GENUS	categorical	—	—	Genus name
15	SPECIES	categorical	—	—	Species name
16	COMMON_NAME	categorical	—	—	Species common name
17	LEAF_PSA	numerical	cm^2^	1–557	Leaf projected surface area (PSA)
18	PSA_to_HSA	numerical	—	1.00–1.57	Coefficient converting PSA to HSA
19	LEAF_HSA	numerical	cm^2^	2–557	Leaf hemisurface area (HSA)
20	LEAF_DRY_WT	numerical	g	0.01–3.88	Leaf sample dry weight
21	LEAF_CARBON_WT	numerical	g C	0.005–1.741	Leaf sample carbon weight
22	SLA_PSA	numerical	cm^2^ PSA g^−1^ C	27–1004	Specific leaf area based on PSA
23	SLA_HSA	numerical	cm^2^ HSA g^−1^ C	43–1,146	Specific leaf area based on HSA
24	LEAF_CARBON	numerical	%	38–57	Leaf carbon mass fraction
25	LEAF_NITROGEN	numerical	%	0.45–3.89	Leaf nitrogen mass fraction
26	LEAF_CN	numerical	—	12–112	Leaf carbon to nitrogen mass ratio
27	LEAF_LIFE	numerical	year	1–26	Leaf lifespan
28	NOTES	categorical	—	—	General notes

**Table 6:** Summary of each column included in the forest carbon cycling dataset, including column number, column name, data format, units, range of values, and a short description.

**Col.**	**Col. name**	**Format**	**Units**	**Range**	**Description**
1	PROJECT	categorical	—	—	Project name
2	YEAR	numerical	year	1999–2014	Sampling year
3	MONTH	numerical	month	5–10	Sampling month
4	DAY	numerical	day	1–31	Sampling day
5	ECOREGION	categorical	—	—	EPA Level III Ecoregion
6	CLUSTER	categorical	—	—	General sampling area
7	PLOT_ID	numerical	—	1–1,014	Plot identification number
8	PLOT_ID_ALT	numerical	—	—	Alternative PLOT_ID is previously sampled
9	PLOT_ID_AMERIFLUX	categorical	—	—	AmeriFlux identification
10	LATITUDE	numerical	decimal degree	37.7844–45.9477	Plot latitude (WGS84)
11	LONGITUDE	numerical	decimal degree	−123.9038–−117.1309	Plot longitude (WGS84)
12	ELEVATION	numerical	m	138–2,758	Elevation above sea level
13	MAT	numerical	°C	2.43–13.53	Mean annual temperature (1984–2013)
14	MAP	numerical	mm yr^−1^	248–2,839	Mean annual precipitation (1984–2013)
15	SITE_DESC	categorical	—	—	General site description
16	ASA	numerical	year	8–795	Average stand age
17	SPP_O1_ ABBREV	categorical	—	—	Primary overstory species abbreviation
18	SPP_O1_BASAL_AREA_FRACTION	numerical	%	33–100	Primary overstory species basal area fraction
19	SPP_O2_ ABBREV	categorical	—	—	Secondary overstory species abbreviation
20	SPP_O2_ BASAL_AREA_FRACTION	numerical	%	0–50	Secondary overstory species basal area fraction
21	SPP_O3_ ABBREV	categorical	—	—	Tertiary overstory species abbreviation
22	SPP_O3_ BASAL_AREA_FRACTION	numerical	%	0–30	Tertiary overstory species basal area fraction
23	SPP_O4_ ABBREV	categorical	—	—	Quaternary overstory species abbreviation
24	SPP_O4_ BASAL_AREA_FRACTION	numerical	%	0–17	Quaternary overstory species basal area fraction
25	LAI_O	numerical	m^2^ m^−2^	0–14.7	Stand overstory leaf area index
26	HEIGHTC	numerical	m	1–54	Average canopy height
27	AG_BIOMASS_TREE_WOOD_AS_CARBON	numerical	g C m^−2^	71–64,035	Tree wood aboveground biomass
28	AG_BIOMASS_TREE_FOLIAGE_AS_CARBON	numerical	g C m^−2^	0–1,738	Tree foliage aboveground biomass
29	AG_BIOMASS_TREE_TOTAL_AS_CARBON	numerical	g C m^−2^	53–65,151	Tree total aboveground biomass
30	AG_PROD_TREE_WOOD_AS_CARBON	numerical	g C m^−2^ yr^−1^	0–800	Tree wood aboveground productivity
31	AG_PROD_TREE_FOLIAGE_AS_CARBON	numerical	g C m^−2^ yr^−1^	0–388	Tree foliage aboveground productivity
32	AG_PROD_TREE_TOTAL_AS_CARBON	numerical	g C m^−2^ yr^−1^	0–958	Tree total aboveground productivity

**Table 7:** Summary of each column included in the soil dataset, including column number, column name, data format, units, range of values, and a short description.

**Col.**	**Col. name**	**Format**	**Units**	**Range**	**Description**
1	PROJECT	categorical	—	—	Project name
2	YEAR	numerical	year	2001–2007	Sampling year
3	MONTH	numerical	month	6–10	Sampling month
4	DAY	numerical	day	1–31	Sampling day
5	ECOREGION	categorical	—	—	EPA Level III Ecoregion
6	CLUSTER	categorical	—	—	General sampling area
7	PLOT_ID	numerical	—	1–948	Plot identification number
8	PLOT_ID_ALT	numerical	—	—	Alternative PLOT_ID is previously sampled
9	PLOT_ID_AMERIFLUX	categorical	—	—	AmeriFlux identification
10	LATITUDE	numerical	decimal degree	37.784–45.948	Plot latitude (WGS84)
11	LONGITUDE	numerical	decimal degree	−123.904–−117.1309	Plot longitude (WGS84)
12	ELEVATION	numerical	m	138–2758	Elevation above sea level
13	MAT	numerical	°C	2.43–13.53	Mean annual temperature (1984–2013)
14	MAP	numerical	mm yr^−1^	414–2,839	Mean annual precipitation (1984–2013)
15	SOIL_DEPTH	numerical	cm	21–100	Total soil depth
16	SOIL_LAYER	categorical	—	top/mid/bottom	Soil layer sampled
17	UPPER_DEPTH_OF_SOIL_LAYER	numerical	cm	0–50	Upper depth of the soil layer
18	LOWER_DEPTH_OF_SOIL_LAYER	numerical	cm	3–103	Lower depth of the soil layer
19	BULK_DENSITY_OF_SOIL_LAYER	numerical	kg m^−2^	2–884	Bulk density of the soil layer
20	CARBON_CONTENT_OF_SOIL_LAYEL	numerical	g C m^−2^	10–24,480	Carbon content of the soil layer
21	NITROGEN_CONTENT_OF_SOIL_LAYER	numerical	g N m^−2^	0–1330	Nitrogen content of the soil layer
22	PH_OF_SOIL_LAYER	numerical	—	3.87–7.45	pH of the soil layer
23	VOLUME_FRACTION_OF_SAND_IN_SOIL_LAYER	numerical	%	17–86	Volume fraction of sand in the soil layer
24	VOLUME_FRACTION_OF_SILT_IN_SOIL_LAYER	numerical	%	11–66	Volume fraction of silt in the soil layer
25	VOLUME_FRACTION_OF_CLAY_IN_SOIL_LAYER	numerical	%	1–50	Volume fraction of clay in the soil layer

**Table 8:** Comparison of leaf trait measurements presented in this study (mean±1s.d.) against estimates drawn from the literature.

**Trait**	**Taxa**	**This study**	**Literature**	**% Diff.**	**Literature sources**
LEAF_LIFE	*Abies lasiocarpa*	10.1±5.0	8.0	23	Reich *et al.*^38^
	*Picea engelmannii*	7.2±2.5	7.5	−4	Reich *et al.*^38^
	*Pinus contorta*	8.0±4.1	2.0, 4.6	120, 67	Pease^40^; Ewers & Schmid^39^
	*Pseudotsuga menziesii*	5.4±2.0	5.4	0	Wright *et al.*^41^
	*Thuja plicata*	5.0	8.9	−56	Harlow *et al.*^42^
	*Tsuga heterophylla*	5.2±1.3	5.4, 5.5	−4, −6	Ishii *et al.*^44^; Pease^40^
	Evergreen needleleaf tree	6.1±3.0	3.3	60	Kattge *et al.*^45^
	Deciduous broadleaf tree	1.4±1.2	0.5	95	Kattge *et al.*^45^
LEAF N	*Acer macrophyllum*	2.81±0.78	2.82	0	Lei & Lechowicz^50^
	*Abies grandis*	0.88±0.16	0.90	−2	Nippert *et al.*^53^
	*Larix occidentalis*	1.87±0.37	1.7, 2.0	10, −7	Gower & Richards^47^; Gower *et al.*^46^
	*Pinus contorta*	1.06±0.21	1.2, 1.4	−12, −28	Gower & Richards^47^; Gower *et al.*^46^
	*Pseudotsuga menziesii*	1.08±0.19	0.99	9	Nippert *et al.*^53^
	*Tsuga mertensiana*	0.89±0.21	1.2	−30	Gower & Richards^47^
	Evergreen needleleaf tree	1.04±0.21	1.21	−15	Kattge *et al.*^45^
	Deciduous broadleaf tree	2.12±0.74	2.13	0	Kattge *et al.*^45^
SLA_HSA*	*Alnus rubra*	326±136	209	44	Matson *et al.*^49^
	*Abies concolor*	102±18	86	17	Laughlin *et al.*^48^
	*Abies grandis*	91±19	95, 112	−4, −21	Nippert *et al.*^53^; Gower & Richards^47^
	*Abies lasiocarpa*	98±13	88	11	Laughlin *et al.*^48^
	*Juniperus occidentalis*	49±2	57	−15	Matson *et al.*^49^
	*Larix occidentalis*	309±33	201, 222	42, 33	Gower & Richards^47^; Gower *et al.*^46^
	*Picea engelmannii*	94±30	76, 116	21, −21	Laughlin *et al.*^48^; Barr *et al.* (2013)
	*Pinus contorta*	80±10	96, 98, 103	−18, −20, −25	Barclay & Goodman^22^; Gower & Richards^47^; Gower *et al.*^46^
	*Pinus ponderosa*	81±11	85, 88	−5, −8	Matson *et al.*^49^; Laughlin *et al.*^48^
	*Pseudotsuga menziesii*	120±24	87, 104, 124, 128, 129	32, 14, −3, −6, −7	Nippert *et al.*^53^; Gower *et al.*^46^; Barclay & Goodman^22^; Ishii *et al.*^43^; Matson *et al.*^49^
	*Thuja plicata*	95±18	167	−55	Barclay & Goodman^22^
	*Tsuga heterophylla*	175±46	132, 316	28, −57	Barclay & Goodman^22^; Ishii *et al.*^44^
	*Tsuga mertensiana*	193±61	68, 104	60	Matson *et al.*^49^; Gower & Richards^47^
	Evergreen needleleaf tree	102±36	108	−6	Kattge *et al.*^45^
	Deciduous broadleaf tree	366±211	324	12	Kattge *et al.*^45^
Traits include leaf lifespan (years), leaf nitrogen (% of dry weight), and specific leaf area (cm^2^ HSA g C^−1^). Trait values drawn from the literature represented mean characteristics. We ordered the literature values and sources numerically for species x trait combinations with multiple literature estimates.					
Estimates of specific leaf area derived from the literature were converted from projected surface area (PSA) per gram of leaf dry matter to hemisurface area (HSA) per gram of carbon using our species-specific measurements of leaf carbon content and the PSA to HSA conversion coefficients given in Table 4.					
